# Dinotefuran aggravates Alzheimer's disease-associated microglial inflammation through the CHUK/NFKB1-mediated NF-κB signaling pathway

**DOI:** 10.1016/j.neurot.2026.e01117

**Published:** 2026-11-04

**Authors:** Gaowa Baibujiafu, Yuwan Lin, Lei Zhang, Lei Du, Shan Yang, You'e Yu, Kaisai Ainiwaer, Jinjing Luo, Xiaobei Wang

**Affiliations:** a Department of Neurology, The Second Affiliated Hospital of Xinjiang Medical University, Urumqi, Xinjiang, 830063, China; b Department of Clinical Psychology, The Fourth People's Hospital of Urumqi, Urumqi, Xinjiang, 830002, China; c Department of Neurology, The First Affiliated Hospital of Guangzhou Medical University, Guangzhou, 510120, Guangdong, China; d Xinjiang Key Laboratory of Neurological Disorder Research, Urumqi, Xinjiang, 830063, China; e Department of Geriatrics, The First Affiliated Hospital of Xinjiang Medical University, Urumqi, Xinjiang, 830011, China

**Keywords:** Dinotefuran, Neonicotinoid pesticides, Alzheimer's disease, Microglia, NF-κB signaling

## Abstract

Neonicotinoid pesticides are widely used systemic insecticides with increasing evidence of neurotoxicity. However, their potential contribution to Alzheimer's disease (AD)-related neuroinflammation remains poorly understood. In this study, seven representative neonicotinoid pesticides were systematically evaluated by integrating physicochemical characterization, target prediction, pathway enrichment analysis, human AD snRNA-seq, molecular docking, molecular dynamics simulations, and experimental validation. Among the seven compounds, dinotefuran was prioritized based on its favorable blood-brain barrier permeability and enrichment in inflammation-related pathways. Integration of target prediction with AD transcriptomic analyses identified CHUK and NFKB1 as key candidate targets associated with NF-κB signaling. snRNA-seq analysis further demonstrated preferential enrichment of NF-κB signaling activity in microglia from AD brains. Molecular docking and molecular dynamics simulations supported potential interactions between dinotefuran and IKKα (CHUK) as well as NF-κB1 (p50). In Aβ-treated BV2 microglia, dinotefuran further enhanced activation of the CHUK/NFKB1-mediated NF-κB signaling pathway, accompanied by increased expression of IKKα, phosphorylation of IκBα and p65, enhanced NF-κB1 activation, and elevated secretion of IL-1β, TNF-α, IL-6, IFN-α, and IFN-β. These pro-inflammatory effects were markedly attenuated by the NF-κB inhibitor BAY11-7082. Collectively, our findings suggest that CHUK/NFKB1-associated NF-κB signaling may contribute to dinotefuran-enhanced inflammatory responses in Aβ-treated BV2 microglia, providing preliminary mechanistic insights into the potential involvement of neonicotinoid pesticides in AD-related neuroinflammation.

## Introduction

Neonicotinoid pesticides are a class of synthetic insecticides structurally analogous to nicotine that primarily exert their insecticidal effects by targeting nicotinic acetylcholine receptors (nAChRs) in the insect nervous system [1], [2]. They are among the most widely used systemic insecticides in modern agriculture because of their high insecticidal efficacy and relatively broad-spectrum activity against agricultural pests [3]. Common neonicotinoid compounds, including imidacloprid, thiamethoxam, clothianidin, acetamiprid, and dinotefuran, are extensively applied in crop protection, seed treatment, and pest management worldwide [4]. Due to their widespread agricultural application and environmental persistence, neonicotinoids have become ubiquitous environmental contaminants that can be detected in soil, surface water, food products, and human biological samples [5], [6], [7], [8]. Increasing evidence suggests that the toxicological effects of neonicotinoids are not restricted to insects but may also affect mammalian physiological systems, particularly the nervous system [9]. Experimental studies have demonstrated that neonicotinoid exposure can induce oxidative stress, neuroinflammatory responses, synaptic dysfunction, and behavioral abnormalities [10], [11], [12]. However, despite growing attention toward the neurotoxicity of neonicotinoids, their potential contribution to Alzheimer's disease (AD)-related neuroinflammation and the underlying molecular mechanisms remain poorly understood.

AD is the most common neurodegenerative disorder and is characterized by progressive cognitive decline, synaptic dysfunction, neuronal loss, and chronic neuroinflammation [13]. Although aging remains the strongest risk factor for AD, increasing evidence suggests that environmental exposures also contribute substantially to disease initiation and progression [14]. Among environmental toxicants, pesticide exposure has attracted considerable attention because of its potential association with neurodegenerative disorders [15], [16]. Previous studies have primarily focused on organophosphate and organochlorine pesticides, which have been linked to oxidative stress, mitochondrial dysfunction, and neuroinflammatory responses in the central nervous system [17], [18]. In contrast, the potential contribution of neonicotinoid pesticides to AD-related pathological processes remains relatively underexplored.

Notably, neonicotinoids act on nicotinic acetylcholine receptors, which are closely associated with synaptic transmission and cognitive function [19], [20]. Dysregulation of cholinergic signaling has long been implicated in AD pathogenesis [21], suggesting that neonicotinoids may influence neurodegenerative processes through neuronal mechanisms. However, it remains unclear which neonicotinoid compounds are most strongly associated with AD-related biological pathways and whether they contribute to neuroinflammatory signaling in the AD brain.

Neuroinflammation is increasingly recognized as a central contributor to AD progression, with microglia serving as the primary immune effector cells in the central nervous system [22]. Under pathological conditions, activated microglia can produce pro-inflammatory cytokines, reactive oxygen species, and inflammasome-related mediators, thereby contributing to synaptic impairment and neuronal injury [23]. Among the key inflammatory pathways, NF-κB signaling plays a pivotal role in regulating microglial activation and inflammatory gene expression [24]. Aberrant activation of NF-κB signaling has been widely implicated in AD-related neuroinflammation and may amplify inflammatory cascades within the brain microenvironment [25]. Therefore, exploring whether neonicotinoid pesticides are associated with microglia-mediated NF-κB signaling may provide mechanistic insight into their potential contribution to AD-related neuroinflammatory processes.

Recent advances in single-nucleus RNA sequencing (snRNA-seq) technologies have provided unprecedented opportunities to characterize cell-type-specific transcriptional alterations in neurodegenerative diseases [26]. Compared with conventional bulk transcriptomic approaches, snRNA-seq enables the resolution of cellular heterogeneity within complex brain tissues and facilitates the investigation of molecular changes within specific cell populations [27]. In particular, single-nucleus transcriptomic analyses have substantially advanced our understanding of AD pathogenesis by revealing disease-associated cellular states, signaling pathways, and intercellular communication networks at unprecedented resolution [28]. In addition, pathway activity scoring approaches based on single-cell transcriptomic data have emerged as powerful tools for evaluating dynamic signaling alterations across distinct cell types [29]. However, the cell-type-specific molecular mechanisms potentially linking neonicotinoid exposure to AD-related inflammatory signaling remain largely unclear.

In the present study, we employed an integrative strategy combining physicochemical characterization, blood-brain barrier permeability prediction, target-based pathway analysis, single-nucleus transcriptomic profiling, molecular docking, molecular dynamics simulation, and experimental validation to systematically investigate the potential association between neonicotinoid pesticides and AD-related neuroinflammation. By integrating computational prediction, transcriptomic profiling, and experimental approaches, this study aimed to explore the potential inflammatory signaling pathways and cell-type-specific mechanisms associated with neonicotinoid exposure in AD. Our findings may provide mechanistic insight into the potential contribution of environmentally relevant neonicotinoid pesticides to AD-related neuroinflammatory processes.

## Methods

### Chemical information and physicochemical characterization of neonicotinoid pesticides

The chemical structures of seven representative neonicotinoid pesticides, including acetamiprid, clothianidin, dinotefuran, imidacloprid, nitenpyram, thiamethoxam, and thiacloprid, were retrieved from the PubChem database (http://pubchem.ncbi.nlm.nih.gov/) in SDF format. These compounds were selected based on their widespread agricultural application, environmental relevance, and representative structural diversity among commonly used neonicotinoid insecticides. The selected compounds covered major neonicotinoid subclasses, including nitroguanidine- and cyanoamidine-based neonicotinoids.

Key physicochemical parameters were calculated using SwissADME (http://www.swissadme.cn/). Radar plots were subsequently generated to visualize and compare the physicochemical profiles of the seven neonicotinoid compounds.

### Prediction of neonicotinoid-associated target genes

Predicted target genes associated with the seven neonicotinoid pesticides were obtained using the TargetNet database (http://targetnet.scbdd.com/). Canonical SMILES of each compound were used as input for target prediction. Predicted targets for each neonicotinoid compound were collected and integrated after duplicate removal. The final target lists were retained for downstream analyses, and predicted target genes are provided in Table S1.

To evaluate the potential association between neonicotinoid-associated targets and AD, gene-disease relevance scores were retrieved from the GeneCards database (https://www.genecards.org/). Relevance scores of AD-associated genes were assigned to the predicted target genes, and cumulative relevance scores were calculated by summing the scores of all associated targets for each compound. These cumulative scores were subsequently used to compare the relative AD relevance among the seven neonicotinoid pesticides.

### Functional enrichment analysis

To characterize the biological relevance of neonicotinoid-associated target genes and transcriptomic alterations, Gene Ontology (GO) enrichment analysis and Kyoto Encyclopedia of Genes and Genomes (KEGG) pathway enrichment analysis were performed using the R package clusterProfiler.

For target-based enrichment analysis, predicted target genes associated with each neonicotinoid compound were used as input for pathway analysis. For transcriptomic analyses, differentially expressed genes (DEGs) identified from single-nucleus datasets were subjected to GO and KEGG enrichment analyses. Pathways and GO terms with p-values <0.05 were considered significantly enriched and were retained for downstream visualization and interpretation.

### Single-nucleus RNA sequencing data processing

Single-nucleus RNA sequencing (snRNA-seq) data derived from the human middle temporal gyrus were downloaded from the Gene Expression Omnibus (GEO) database under accession number GSE188545, comprising six healthy control (HC) samples and six AD samples. Data preprocessing and downstream analyses were performed using the Seurat R package.

Quality control was conducted to remove low-quality nuclei. Nuclei with fewer than 300 detected genes or more than 5000 detected genes, as well as nuclei with mitochondrial gene percentages greater than 10%, were excluded from subsequent analyses. After filtering, gene expression data were normalized using the NormalizeData function, and highly variable genes were identified using the FindVariableFeatures function with the “vst” method. Data scaling was subsequently performed using the ScaleData function.

Principal component analysis (PCA) was applied for dimensionality reduction, and significant principal components were selected for downstream analyses. Uniform manifold approximation and projection (UMAP) was used for visualization, and graph-based clustering was performed using Seurat functions.

Cell-type annotation was conducted based on established canonical marker genes, including neurons (SYT1 and SNAP25), astrocytes (AQP4 and SLC1A3), microglia (ITGAM and CD74), oligodendrocytes (MBP and MOBP), oligodendrocyte precursor cells (VCAN and PDGFRA), and vascular cells (FLT1 and DCN).

### Pathway activity scoring analysis

To quantify pathway activity at the single-nucleus level, gene set-based enrichment analysis was performed using the irGSEA R package. KEGG pathway gene sets were used as the reference collection for pathway scoring.

To improve analytical robustness, four independent scoring algorithms, including AUCell, UCell, singscore, and ssGSEA, were applied in parallel to calculate pathway activity scores based on normalized gene expression data. Pathway activity differences among distinct cell populations were subsequently compared according to cell-type annotations.

To integrate results from different scoring approaches, Robust Rank Aggregation (RRA) analysis was performed to obtain consensus pathway enrichment rankings across algorithms. Pathways with adjusted p-value ≤0.05 were considered significantly dysregulated and were retained for downstream analyses and visualization.

### Molecular docking

Molecular docking was performed to evaluate the potential binding interactions between dinotefuran and NF-κB-associated target proteins, including IKKα encoded by CHUK and NF-κB p50 encoded by NFKB1. Protein structures were obtained from the Protein Data Bank, and the dinotefuran structure was retrieved from PubChem. Protein and ligand preparation, receptor grid generation, and docking simulations were conducted using the Schrödinger software package. Docking was performed using Glide extra precision mode, and protein–ligand binding conformations and interaction patterns were visualized using Maestro.

### Molecular dynamics simulation

To assess the dynamic stability of the docked protein–ligand complexes, 150 ns molecular dynamics simulations were performed using the Desmond module in the Schrödinger software package. The docking-derived conformations were used as the initial structures for simulation. Simulation trajectories were analyzed using the Simulation Interaction Diagram module. Root mean square deviation, root mean square fluctuation, radius of gyration, and hydrogen bond interactions were calculated to evaluate the structural stability and dynamic behavior of each complex.

### Cell culture and dinotefuran exposure

BV2 microglial cells were cultured in Dulbecco's modified Eagle's medium (DMEM; Gibco, USA) supplemented with 10% fetal bovine serum (FBS; Gibco, USA) and 1% penicillin–streptomycin at 37 °C in a humidified incubator containing 5% CO_2_.

Dinotefuran (sc-200615, Santa Cruz Biotechnology, USA) was dissolved in dimethyl sulfoxide (DMSO) to prepare a stock solution and diluted with culture medium to the desired concentrations before use. Amyloid-β_1-42_ (Aβ_1-42_; A834109, Macklin, Shanghai, China) was used to establish an in vitro AD-associated inflammatory model. To investigate the effects of dinotefuran on Aβ-induced inflammatory responses, BV2 cells were randomly assigned to the following groups: control, Aβ, dinotefuran, Aβ + dinotefuran, and Aβ + dinotefuran + BAY11-7082. BAY11-7082 was used as a pharmacological inhibitor of canonical NF-κB signaling [30]. All treatments were performed for 24 h. Following the indicated treatments, cells and culture supernatants were collected for subsequent western blotting, immunofluorescence staining, and ELISA analyses.

### Cell viability assay

BV2 cell viability was assessed using a Cell Counting Kit-8 (CCK-8) assay (Cat. No. CA1210, Solarbio, Beijing, China) according to the manufacturer's instructions. Briefly, BV2 cells were seeded into 96-well plates and exposed to increasing concentrations of dinotefuran for 24 h. Subsequently, 10 μL of CCK-8 solution was added to each well and incubated at 37 °C for 1–2 h.

Absorbance was measured at 450 nm using a microplate reader, and cell viability was calculated relative to the control group. Dose–response curves were generated to evaluate the cytotoxic effects of dinotefuran and to determine the concentration used for subsequent experiments.

### ELISA

The concentrations of IL-1β (Cat. No. 88–7013), IL-6 (Cat. No. 88–7064), and TNF-α (Cat. No. 88–7324) were measured using ELISA kits purchased from Thermo Fisher Scientific (Waltham, MA, USA), whereas IFN-α (Cat. No. EK199) and IFN-β (Cat. No. EK2236-01) were quantified using ELISA kits obtained from MultiSciences Biotech Co., Ltd. (Hangzhou, China). Cell culture supernatants were analyzed according to the manufacturers’ instructions. Absorbance was measured using a microplate reader, and cytokine concentrations were calculated based on standard curves generated using recombinant standards supplied with each kit.

### Western blot analysis

Total protein was extracted from BV2 cells using RIPA lysis buffer supplemented with protease and phosphatase inhibitors. Protein concentrations were quantified using a BCA protein assay kit according to the manufacturer's instructions.

Equal amounts of protein were separated by SDS-PAGE and subsequently transferred onto PVDF membranes. After blocking with 5% non-fat milk for 1 h at room temperature, the membranes were incubated overnight at 4 °C with primary antibodies against IKKα (F0995, Selleck Chemicals), NF-κB1 p105/p50 (F0439, Selleck Chemicals), phospho–NF–κB p65 (Ser 536) (3033 S, Cell Signaling Technology), NF-κB p65 (10745-1-AP, Proteintech), phospho-IκBα (82349-1-RR, Proteintech), IκBα (10268-1-AP, Proteintech), and GAPDH (60004-1-Ig, Proteintech). Membranes were subsequently incubated with horseradish peroxidase (HRP)-conjugated secondary antibodies for 1 h at room temperature.

Protein bands were visualized using an enhanced chemiluminescence (ECL) detection system and quantified using ImageJ software. GAPDH was used as the internal loading control.

### Immunofluorescence staining

BV2 cells were fixed with 4 % paraformaldehyde, permeabilized using Triton X-100, and blocked with bovine serum albumin. Cells were subsequently incubated overnight at 4 °C with primary antibodies against microglial and NF-κB-related markers, followed by incubation at room temperature with fluorochrome-conjugated secondary antibodies. Nuclei were counterstained with DAPI.

Fluorescence images were captured using a confocal fluorescence microscope under identical imaging settings across experimental groups. Fluorescence intensity was quantified using ImageJ software.

### Statistical analysis

All statistical analyses were performed using GraphPad Prism and R software. Data are presented as mean ± standard deviation (SD). Comparisons between two groups were performed using unpaired Student's t-tests, while comparisons among multiple groups were analyzed using one-way analysis of variance (ANOVA) followed by appropriate post hoc tests.

For bioinformatic analyses, adjusted p-values generated from differential expression and enrichment analyses were used for significance evaluation. A p-value <0.05 was considered statistically significant.

## Results

### Blood-brain barrier permeability prediction and physicochemical characterization of neonicotinoid pesticides

The chemical structures and physicochemical properties of seven commonly used neonicotinoid pesticides, including Acetamiprid, Clothianidin, Dinotefuran, Imidacloprid, Nitenpyram, Thiamethoxam, and Thiacloprid, were first characterized (Fig. 1A–G). Multiple physicochemical parameters, including molecular weight (MW), hydrogen bond acceptors (HBA), hydrogen bond donors (HBD), dipole moment, solvent-accessible surface area (SASA), fractional hydrophobic surface area (FISA), fractional polar surface area (FOSA), and polar surface area (PISA), were analyzed using radar plots. Distinct physicochemical profiles were observed among the seven neonicotinoid compounds, and differences in these parameters may determine their distinct physicochemical characteristics and BBB permeability potentials.

**Fig. 1:**
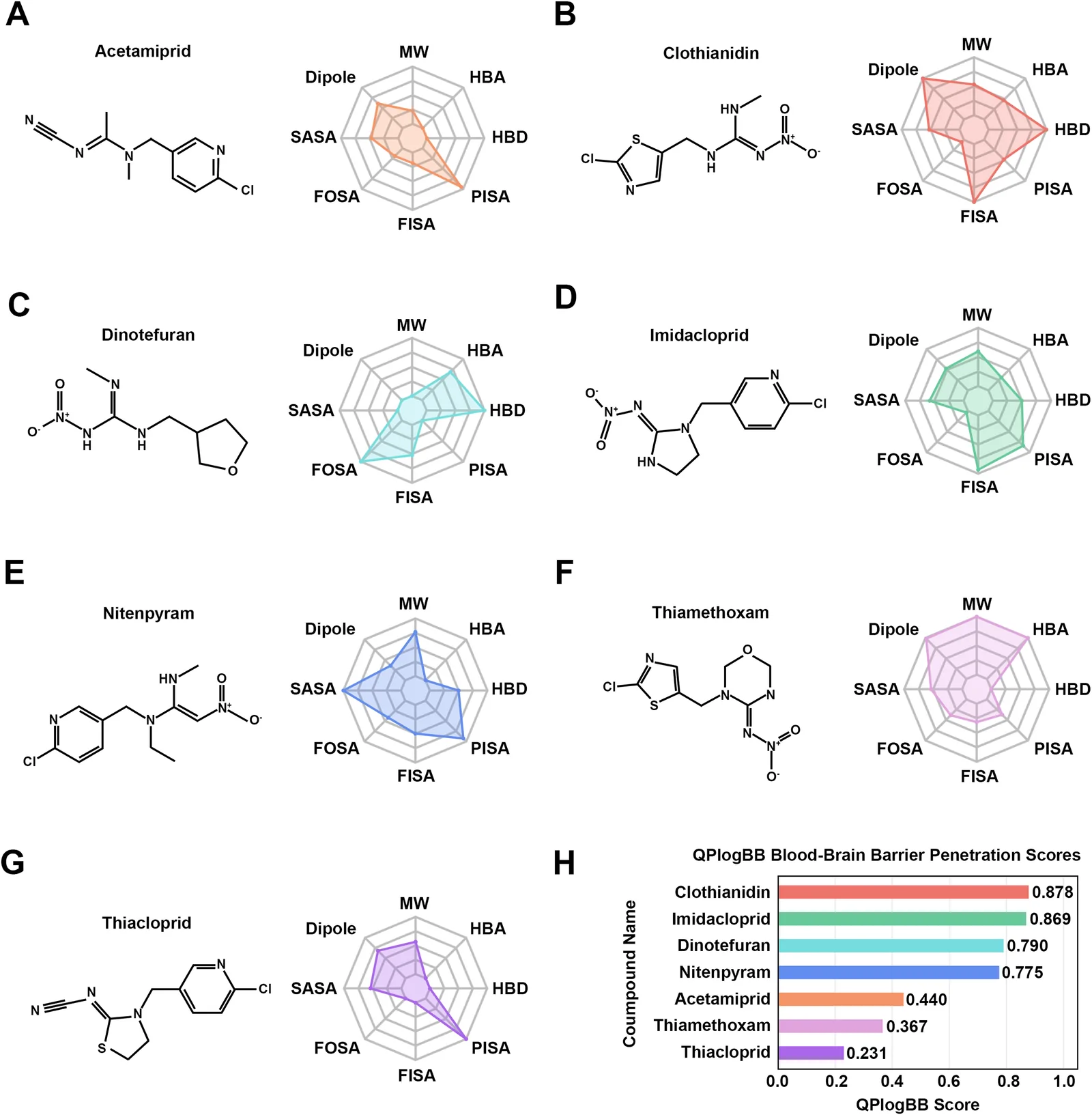
**Physicochemical properties and predicted blood-brain barrier permeability of representative neonicotinoid insecticides.** (A–G) Chemical structures and radar plots illustrating the physicochemical characteristics of acetamiprid (A), clothianidin (B), dinotefuran (C), imidacloprid (D), nitenpyram (E), thiamethoxam (F), and thiacloprid (G). Radar plots summarize molecular weight (MW), hydrogen bond acceptor (HBA), hydrogen bond donor (HBD), polar surface area-related indices (PISA, FISA, FOSA), solvent-accessible surface area (SASA), and dipole moment. (H) Predicted blood-brain barrier penetration potential of the seven neonicotinoids based on QPlogBB scores. Higher QPlogBB values indicate stronger predicted BBB permeability.

To further evaluate their potential central nervous system accessibility, BBB permeability was predicted using in silico pharmacokinetic models. QPlogBB scores indicated that Clothianidin, Imidacloprid, Dinotefuran, and Nitenpyram exhibited relatively high predicted BBB permeability, whereas Acetamiprid, Thiamethoxam, and Thiacloprid showed comparatively lower BBB penetration potential (Fig. 1H).

Collectively, these analyses suggest that several neonicotinoid pesticides may have the potential to penetrate the BBB and exert central nervous system-related effects.

### Integrated target prediction prioritizes dinotefuran as an AD-associated neonicotinoid linked to inflammatory signaling

To further investigate the potential biological relevance of neonicotinoid pesticides in neurodegeneration, predicted target genes associated with the seven neonicotinoids were retrieved from the TargetNet database (Table S1). Comparative analysis revealed both shared and compound-specific target distributions among the neonicotinoid compounds, indicating overlapping yet heterogeneous biological associations (Fig. 2A).

**Fig. 2:**
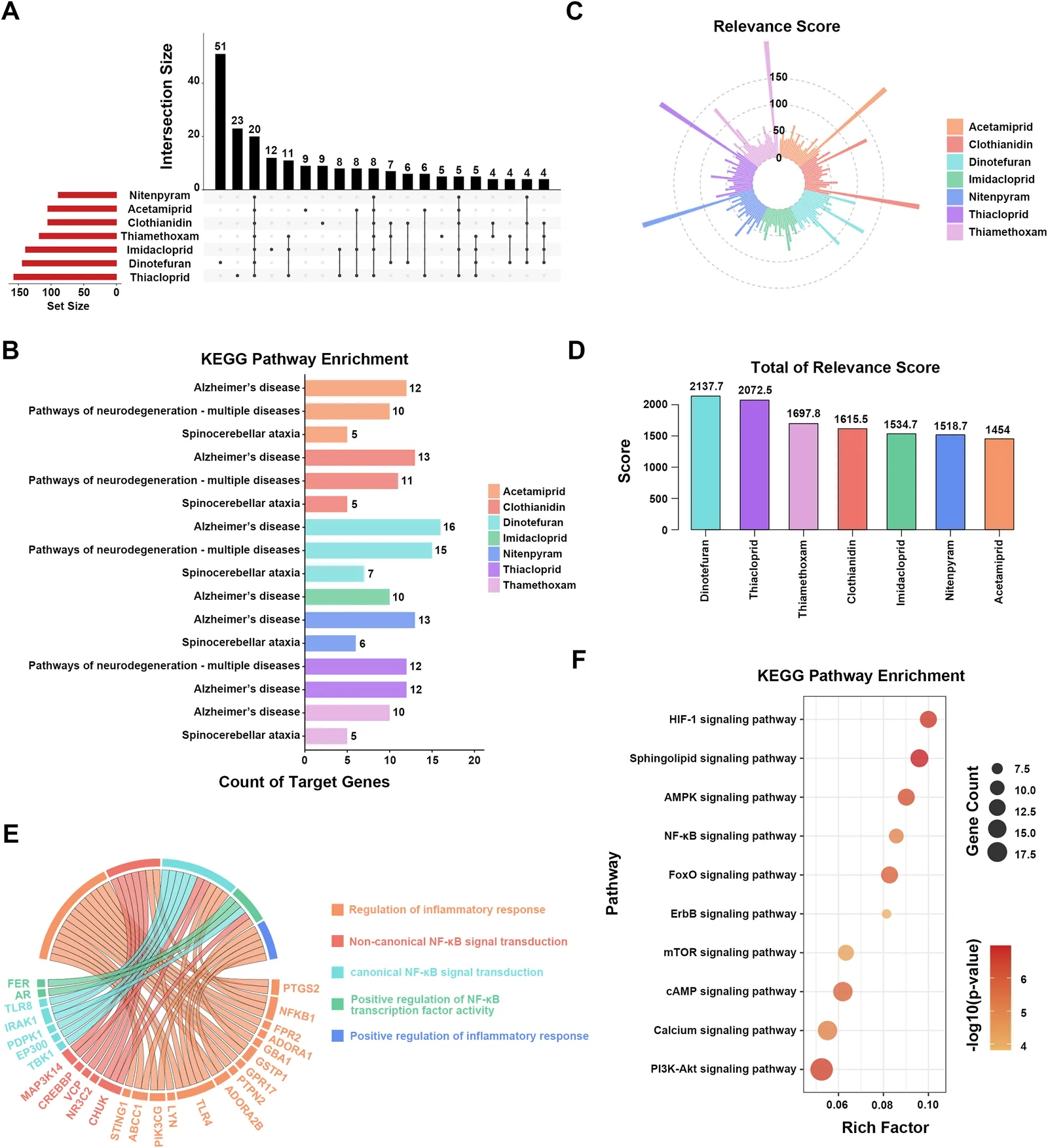
**Network toxicology analysis and neurodegeneration-related pathway enrichment of representative neonicotinoids.** (A) UpSet plot showing the overlap and unique target genes among seven representative neonicotinoid insecticides. (B) KEGG enrichment analysis of compound-associated targets. (C) Circular bar plot displaying AD-related relevance scores of predicted targets for each compound. (D) Total relevance scores of each neonicotinoid were calculated by summing AD-associated target relevance scores. (E) Chord diagram showing the relationship between core target genes and inflammation-related biological processes. (F) KEGG enrichment analysis of shared neuroinflammation-associated targets.

To characterize the disease-related biological pathways potentially affected by neonicotinoid pesticides, KEGG enrichment analysis was performed using the predicted target genes. The enriched pathways included several neurodegeneration-associated pathways, such as AD, pathways of neurodegeneration-multiple diseases, and spinocerebellar ataxia (Fig. 2B), suggesting a potential link between neonicotinoid-associated targets and neurodegenerative processes.

Based on the enrichment of AD-related pathways, AD-associated relevance scores were further calculated for the predicted target genes and visualized for each compound (Fig. 2C). To compare the relative AD relevance among compounds, cumulative values for each neonicotinoid. Among the analyzed compounds, dinotefuran exhibited the highest total relevance score (Fig. 2D), suggesting a relatively stronger association between dinotefuran-related targets and AD-associated biological processes.

Functional enrichment analysis further revealed that dinotefuran-associated targets were predominantly enriched in inflammation-related biological processes and NF-κB-associated signaling pathways (Fig. 2E). Consistently, KEGG pathway enrichment analysis highlighted multiple inflammation- and stress-related pathways, including NF-κB signaling, PI3K-Akt signaling, mTOR signaling, AMPK signaling, calcium signaling, FoxO signaling, sphingolipid signaling, and HIF-1 signaling pathways (Fig. 2F).

Collectively, these findings suggest that dinotefuran is closely associated with neurodegeneration-related and inflammatory signaling pathways, particularly NF-κB-associated signaling, supporting its prioritization for subsequent single-cell transcriptomic and mechanistic validation analyses.

### Single-nucleus transcriptomics reveals preferential NF-κB signaling activation in microglia in AD brain

To investigate the cell-type-specific inflammatory landscape associated with AD, single-nucleus transcriptomic data from human AD brain samples were analyzed. Major brain cell populations, including ASC, MG, NEU, ODC, OPC, and VC, were identified based on established marker genes (Fig. 3A–C). Comparative analysis of cell composition revealed moderate alterations in the relative proportions of several cell populations between HC and AD samples (Fig. 3D–E).

**Fig. 3:**
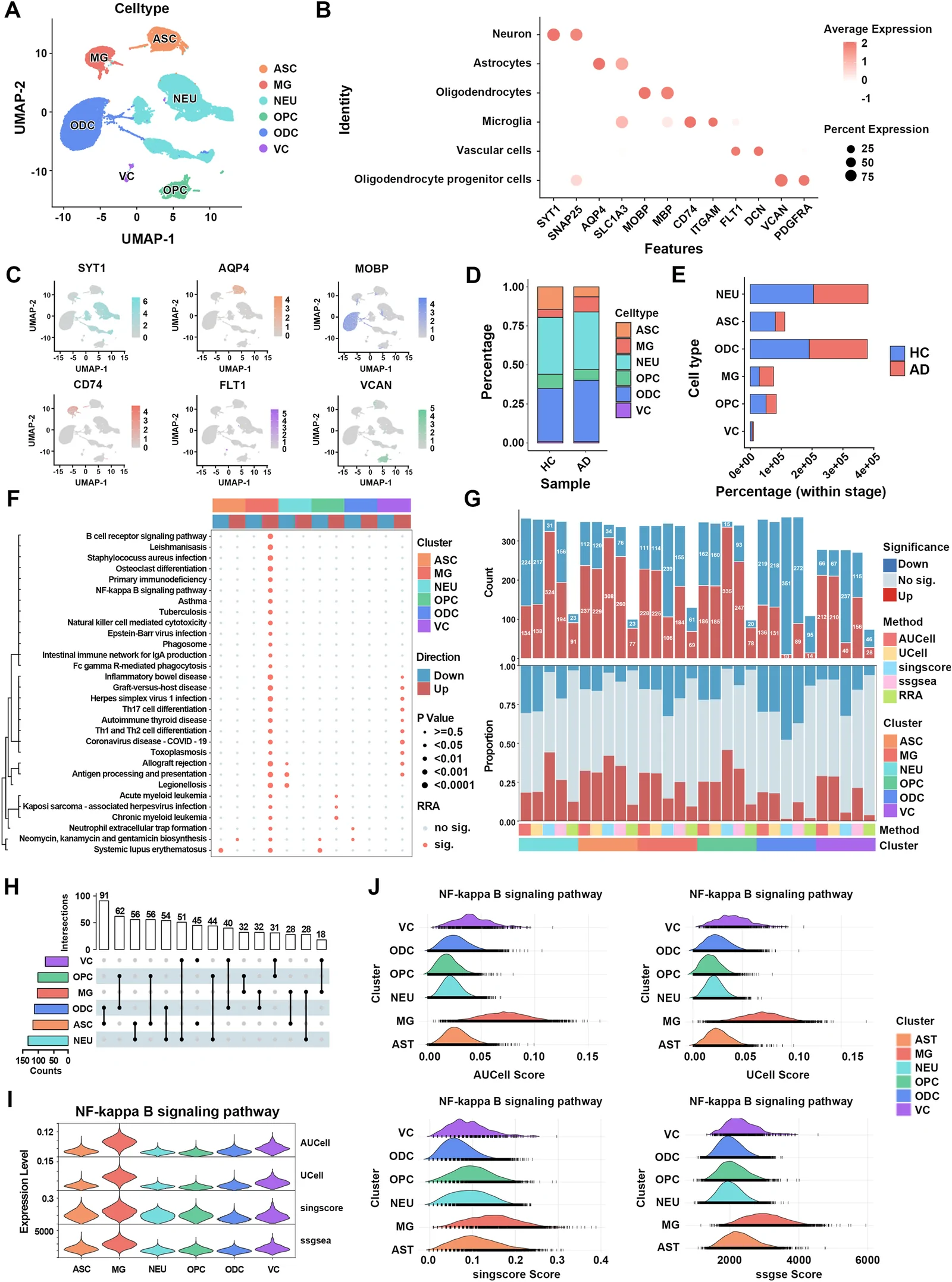
**Single-nucleus transcriptomic profiling and pathway activity analysis reveal microglia-specific NF-κB signaling alterations in Alzheimer's disease.** (A) UMAP visualization of major brain cell populations identified from single-nucleus transcriptomic data, including astrocytes (ASC), microglia (MG), neurons (NEU), oligodendrocytes (ODC), oligodendrocyte precursor cells (OPC), and vascular cells (VC). (B) Dot plot showing canonical marker gene expression across annotated cell populations. (C) Feature plots displaying representative marker genes for major cell populations, including SYT1, AQP4, MOBP, CD74, FLT1, and VCAN. (D) Relative proportions of major cell populations in healthy control (HC) and Alzheimer's disease (AD) samples. (E) Comparative distribution of cell populations between HC and AD groups. (F) Differential pathway enrichment analysis across cell populations using Robust Rank Aggregation (RRA)-integrated pathway scoring. (G) Summary of pathway activity alterations identified by multiple scoring algorithms, including AUCell, UCell, singscore, ssGSEA, and RRA. Upper panels indicate the number of significantly upregulated and downregulated pathways, while lower panels show pathway proportions of enriched pathways among different cell populations. (H) UpSet plot showing overlaps of significantly altered pathways across major brain cell populations. (I) Violin plots comparing NF-κB signaling pathway activity scores across major cell populations using AUCell, UCell, singscore, and ssGSEA methods. (J) Density distributions of NF-κB signaling pathway activity scores across cell populations calculated by AUCell, UCell, singscore, and ssGSEA.

To further characterize pathway activity across distinct cell populations, multiple single-cell pathway scoring algorithms, including AUCell, UCell, singscore, and ssGSEA, were integrated using Robust Rank Aggregation (RRA) analysis. Differential pathway enrichment analysis demonstrated widespread alterations in immune- and inflammation-related signaling pathways across multiple brain cell populations in AD (Fig. 3F–H).

As NF-κB signaling emerged from the dinotefuran target-based enrichment analysis, we further assessed whether this pathway displayed cell-type-specific activation in the AD brain. Multi-algorithm pathway scoring consistently showed that NF-κB signaling activity was most pronounced in microglia compared with other major brain cell populations (Fig. 3I–J). Density distribution and violin plot analyses further demonstrated a clear rightward shift and elevated pathway activity scores in microglia, supporting preferential activation of NF-κB-associated inflammatory signaling within this cell population.

Collectively, these findings indicate that NF-κB signaling is preferentially activated in microglia in the AD brain, providing cell-type-specific transcriptomic evidence supporting the involvement of inflammatory signaling pathways identified in the preceding neonicotinoid target analyses.

### CHUK and NFKB1 are identified as core dinotefuran-associated NF-κb/AD targets

To further identify key molecular targets linking dinotefuran-associated signaling with AD-related neuroinflammation, differential expression analysis was performed in microglia between AD and HC samples. Volcano plot analysis revealed widespread transcriptional alterations in AD microglia, including both significantly upregulated and downregulated genes (Fig. 4A–B). Representative DEGs were further visualized using heatmap analysis, showing distinct expression patterns between AD and HC microglia (Fig. 4C).

**Fig. 4:**
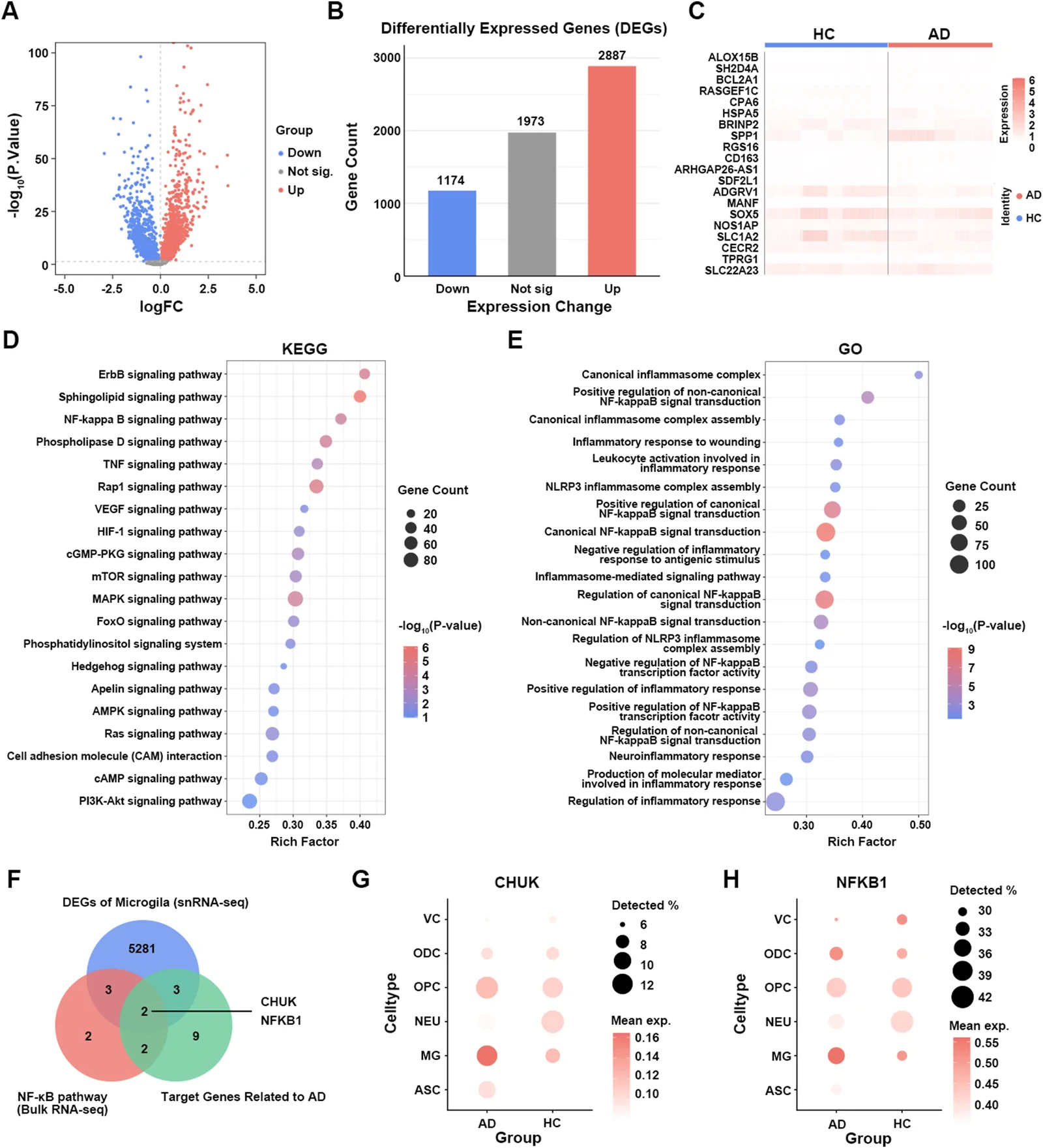
**Microglia-specific differential expression and enrichment analyses identify NF-κB-associated inflammatory signatures in Alzheimer's disease.** (A) Volcano plot showing differentially expressed genes (DEGs) in microglia between AD and HC samples. (B) Summary of DEGs counts in microglia, including upregulated, downregulated, and non-significant genes. (C) Heatmap showing representative DEGs in microglia from HC and AD samples. (D) KEGG pathway enrichment analysis of microglial DEGs. (E) GO enrichment analysis of microglial DEGs. (F) Venn diagram showing the overlap among microglial DEGs identified from snRNA sequencing data, NF-κB pathway-associated genes, and AD-related target genes. (G–H) Dot plots showing the expression patterns of CHUK (G) and NFKB1 (H) across major brain cell populations in HC and AD samples.

Functional enrichment analysis of microglial DEGs demonstrated significant involvement of inflammation- and immune-related pathways. KEGG enrichment analysis highlighted multiple signaling pathways related to inflammatory regulation. Analysis showed enrichment of biological processes associated with inflammatory response, regulation of immune signaling, and NF-κB-related pathways (Fig. 4D). Consistently, GO enrichment analysis showed enrichment of biological processes associated with inflammatory response, regulation of immune signaling, and NF-κB-related pathways (Fig. 4E).

Based on the enrichment of NF-κB signaling in both dinotefuran-associated target analysis and AD microglial transcriptomic analysis, we next intersected three gene sets: dinotefuran-predicted target genes, NF-κB pathway-related genes, and AD microglial differentially expressed genes. This analysis identified CHUK and NFKB1 as shared genes among these datasets (Fig. 4F). CHUK encodes IKKα, a key upstream kinase involved in NF-κB pathway activation, whereas NFKB1 encodes the p50 subunit of the NF-κB transcription factor complex.

To determine the cellular expression patterns of these genes, CHUK and NFKB1 expression was further examined across major brain cell populations. Dot plot analysis showed that both CHUK and NFKB1 were increased in microglia from AD samples compared with HC samples (Fig. 4G–H). These results suggest that CHUK and NFKB1 may serve as key NF-κB-related molecular targets linking dinotefuran-associated target profiles with AD microglial inflammatory activation.

### Molecular docking and dynamics simulations support potential interactions between dinotefuran and CHUK/NFKB1-associated proteins

To further explore the potential molecular interactions between dinotefuran and NF-κB-associated target proteins, molecular docking analysis was performed using IKKα and NF-κB p50. Docking results demonstrated that dinotefuran could be accommodated within the binding pockets of both proteins and formed multiple hydrogen bond interactions with surrounding amino acid residues (Fig. 5A), suggesting favorable binding conformations between dinotefuran and the two NF-κB-related proteins.

**Fig. 5:**
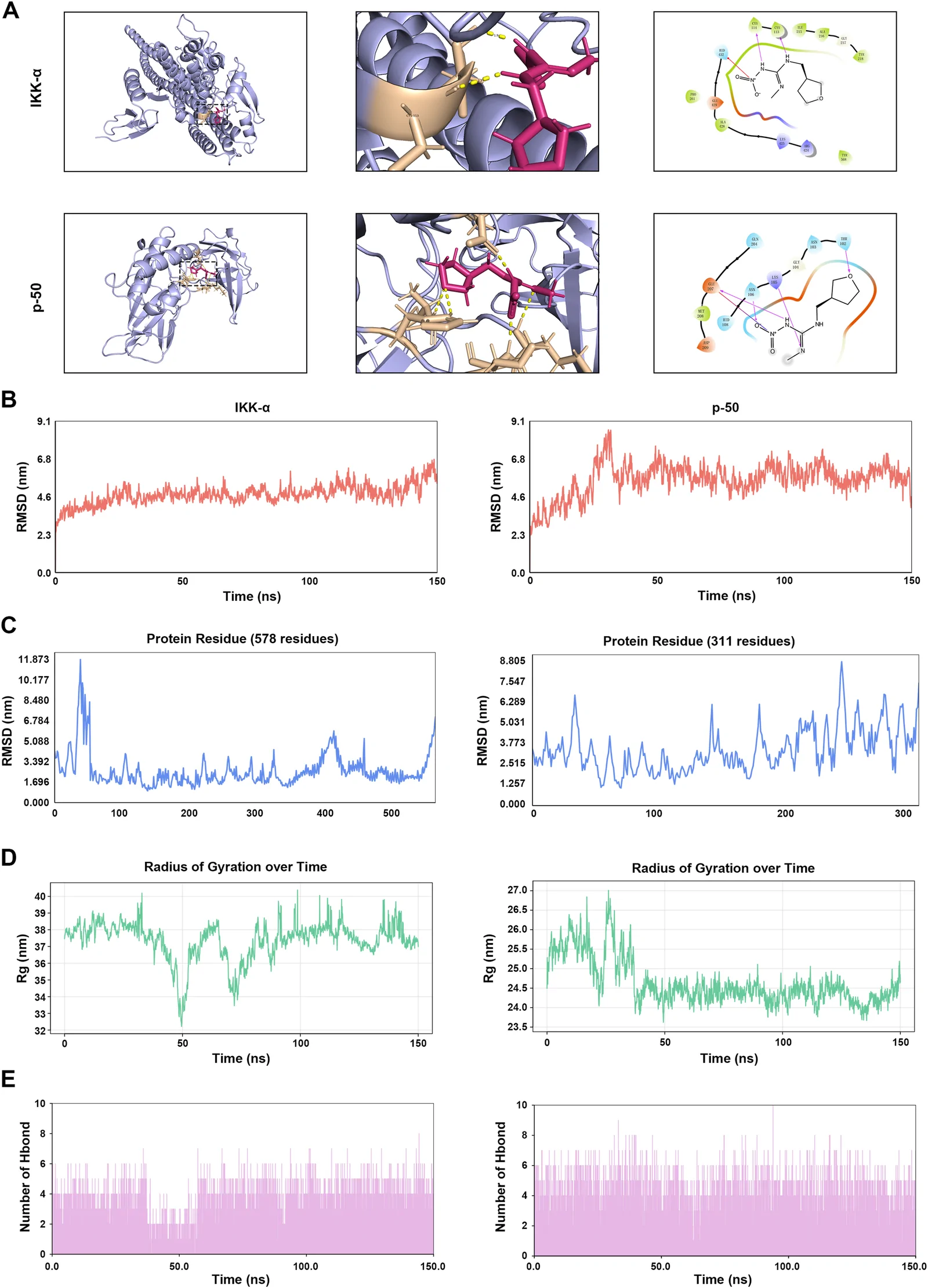
**Molecular docking and molecular dynamics simulation of neonicotinoid interactions with NF-κB-related proteins.** (A) Molecular docking models of representative neonicotinoids binding to IKK-α and NF-κB p50. The left panel shows the overall protein-ligand complexes, the middle panels present detailed binding conformations and hydrogen bond interactions within the binding pockets, and the right panels display two-dimensional interaction diagrams between ligands and surrounding amino acid residues. (B) Root mean square deviation (RMSD) analysis of the protein-ligand complexes during 150 ns molecular dynamics simulations, showing the structural stability of IKK-α-ligand and NF-κB p50-ligand interactions over time. (C) Root mean square fluctuation (RMSF) analysis of amino acid residues in IKK-α and NF-κB p50 during molecular dynamics simulations. Residue-level fluctuations were calculated across the full simulation trajectories. (D) Radius of gyration (Rg) analysis showing changes in structural compactness of the IKKα–dinotefuran and NF-κB p50–dinotefuran complexes over time. (E) Time-dependent hydrogen bond profiles between neonicotinoid compounds and target proteins throughout the molecular dynamics simulations. The number of hydrogen bonds formed at each simulation time point is shown.

To further evaluate the dynamic stability of these protein-ligand complexes, 150 ns molecular dynamics simulations were subsequently performed. Root mean square deviation (RMSD) analysis showed that both the IKKα-dinotefuran and p50-dinotefuran complexes reached relatively stable conformational states following the initial equilibration phase, with limited fluctuations throughout the remainder of the simulations (Fig. 5B), indicating overall structural stability of the complexes.

Residue-level flexibility was further assessed using root mean square fluctuation (RMSF) analysis. Most amino acid residues in both proteins exhibited relatively low fluctuation amplitudes during the simulations, while only several terminal or loop regions showed increased flexibility (Fig. 5C). These results suggest that the simulated protein–ligand complexes maintained relatively stable conformations.

Radius of gyration (Rg) analysis further demonstrated relatively stable structural compactness of the two protein-ligand complexes throughout the simulations (Fig. 5D). In addition, hydrogen bond analysis showed persistent intermolecular hydrogen bond interactions between dinotefuran and both target proteins during the simulation trajectories (Fig. 5E), further supporting the stability of these interactions.

Collectively, molecular docking and molecular dynamics simulations suggest potentially stable interactions between dinotefuran and IKKα or NF-κB1 (p50) under computational conditions, providing a preliminary structural basis for further experimental investigation.

### Dinotefuran aggravates Aβ-induced microglial inflammation through activation of the CHUK/NFKB1–NF-κb signaling pathway

To experimentally validate the predicted CHUK/NFKB1-mediated NF-κB signaling pathway, BV2 microglia were treated with Aβ and dinotefuran. Cell viability was first evaluated using a CCK-8 assay. Dinotefuran exhibited dose-dependent cytotoxicity, and 100 μM was selected for subsequent experiments because it represented a sub-cytotoxic concentration with limited effects on cell viability (Fig. 6A). CHUK encodes IKKα, a key upstream kinase responsible for initiating canonical NF-κB activation through phosphorylation of IκBα [31], whereas NFKB1 encodes the precursor protein p105, which undergoes proteolytic processing to generate the mature p50 subunit of the NF-κB transcription factor complex [32].

**Fig. 6:**
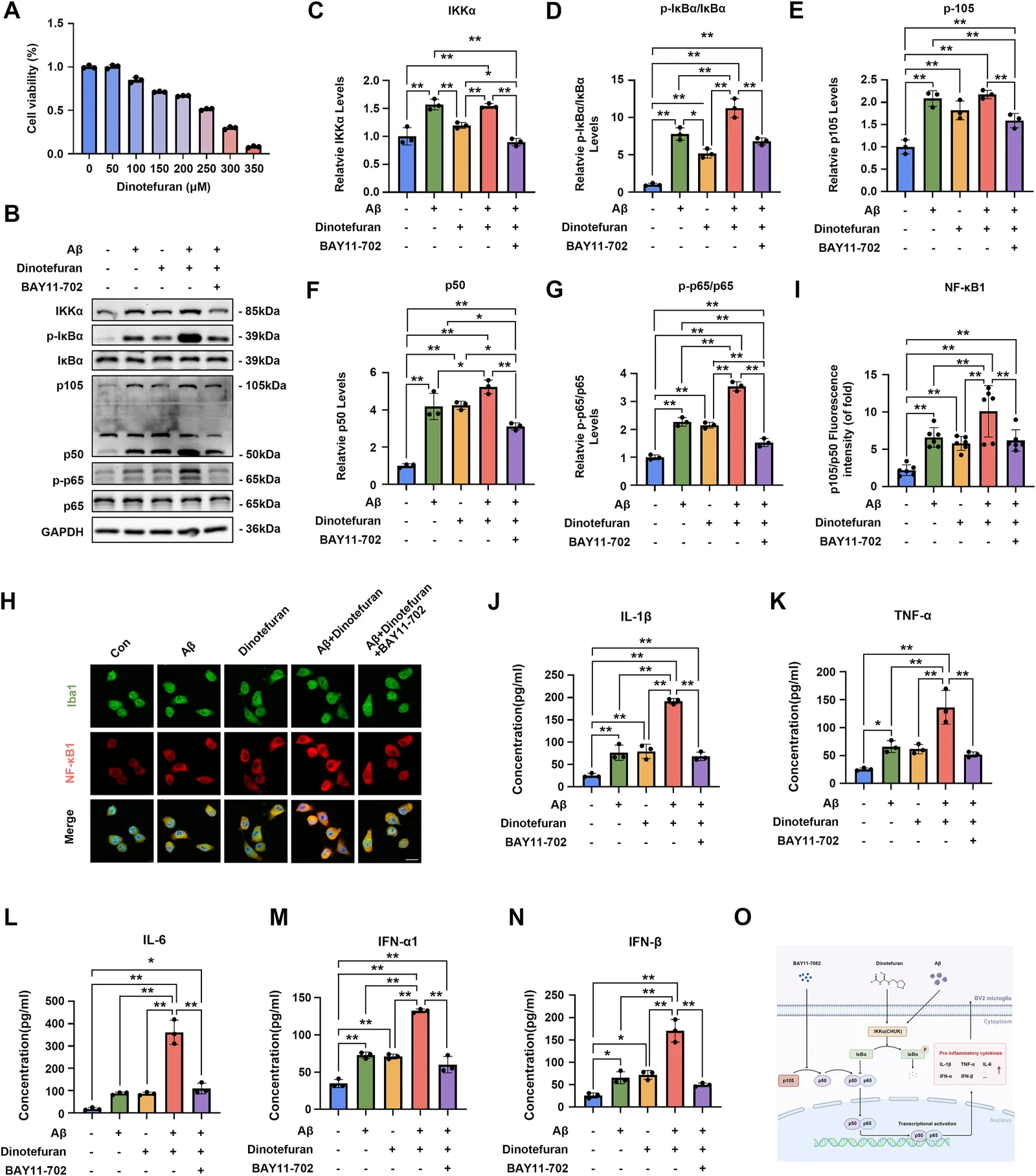
**Dinotefuran exacerbates Aβ-induced NF-κB activation and inflammatory responses in BV2 microglia.** (A) Cell viability of BV2 microglia following 24 h exposure to increasing concentrations of dinotefuran, as determined by CCK-8 assay. (B) Representative Western blot images showing the protein expression levels of IKKα, *p*-IκBα, IκBα, NF-κB1 precursor p105, NF-κB1 mature form p50, p-p65, and p65 in BV2 cells treated with Aβ, dinotefuran, and the NF-κB inhibitor BAY11-7082. (C–G) Quantitative analysis of Western blot results showing the relative protein expression of IKKα (C), the *p*-IκBα/IκBα ratio (D), p105 (E), p50 (F), and the p-p65/p65 ratio (G). (H) Representative immunofluorescence staining of NF-κB1 in BV2 microglia following the indicated treatments. (I) Quantification of NF-κB1 immunofluorescence intensity. (J–N) ELISA analysis of IL-1β (J), TNF-α (K), IL-6 (L), IFN-α (M), and IFN-β (N) levels in the culture supernatants of BV2 cells. (O) Schematic illustration of the proposed mechanism underlying dinotefuran-enhanced Aβ-induced inflammatory responses in BV2 microglia.

Western blot analysis demonstrated that Aβ treatment activated the canonical NF-κB signaling pathway compared with the control group. Compared with Aβ treatment alone, co-treatment with dinotefuran further enhanced NF-κB signaling, as evidenced by significantly increased *p*-IκBα/IκBα and p-p65/p65 ratios, accompanied by increasing trends in IKKα, p105, and p50 expression (Fig. 6B–G). These alterations were markedly attenuated by treatment with the NF-κB inhibitor BAY11-7082. Consistent with the Western blot results, immunofluorescence staining further demonstrated a significant increase in NF-κB1 fluorescence intensity in BV2 microglia following co-treatment with Aβ and dinotefuran, whereas BAY11-7082 effectively suppressed NF-κB1 activation (Fig. 6H and I). Consistent with these findings, ELISA analysis showed that co-treatment with Aβ and dinotefuran significantly increased the concentrations of IL-1β, TNF-α, IL-6, IFN-α, and IFN-β in BV2 cell culture supernatants compared with Aβ treatment alone (Fig. 6J–N). These increases were significantly attenuated by BAY11-7082, further supporting the involvement of NF-κB signaling in dinotefuran-enhanced inflammatory responses.

Collectively, these findings demonstrate that dinotefuran enhances Aβ-induced inflammatory responses in BV2 microglia and that these effects are accompanied by activation of the CHUK/NFKB1-associated NF-κB signaling pathway.

## Discussion

In the present study, we systematically investigated the potential association between neonicotinoid pesticides and AD-related neuroinflammation using an integrative analytical framework. By combining physicochemical characterization, blood-brain barrier permeability prediction, target-based pathway analysis, single-nucleus transcriptomic analysis, molecular docking, molecular dynamics simulation, and experimental validation, dinotefuran was prioritized as a representative neonicotinoid associated with AD-related inflammatory signaling. Single-nucleus transcriptomic analyses demonstrated preferential activation of NF-κB signaling in microglia in the AD brain. In addition, CHUK and NFKB1 were identified as potential NF-κB-associated targets linking dinotefuran-related signaling to AD microglial inflammatory responses, while structural and experimental analyses further supported the potential involvement of dinotefuran in NF-κB-mediated inflammatory activation. Collectively, these findings suggest that dinotefuran may enhance Aβ-associated inflammatory responses in BV2 microglia, although its effects on AD-related neuroinflammation in vivo remain to be established.

Neonicotinoid pesticides are increasingly recognized as emerging environmental neurotoxicants because of their widespread human exposure and potential effects on the central nervous system [33]. Previous studies have demonstrated that neonicotinoid exposure can induce oxidative stress, mitochondrial dysfunction, neuronal injury, and neurobehavioral abnormalities in experimental models [34], [35], [36]. In addition, several neonicotinoid compounds have been reported or predicted to possess blood-brain barrier permeability potential, raising concerns regarding their direct effects on brain homeostasis and neurodegenerative processes [37], [38], [39]. However, compared with traditional pesticide classes such as organophosphates and organochlorines, the potential contribution of neonicotinoids to AD-related pathological processes remains relatively underexplored.

Among the analyzed compounds, dinotefuran was prioritized in the present study because of its relatively high predicted BBB permeability and strong association with AD-related inflammatory pathways. These findings provide population-level evidence supporting the potential relevance of environmentally exposed neonicotinoids in AD-associated biological processes. Although the current study does not establish a direct causal relationship between dinotefuran exposure and AD progression, our results suggest that specific neonicotinoid compounds may possess distinct neuroinflammatory relevance and therefore warrant further investigation in the context of neurodegenerative diseases.

Neuroinflammation is a central pathological feature of AD, and microglia are the principal immune cells responsible for sensing and responding to inflammatory stimuli in the brain [40]. Under AD-related pathological conditions, microglia can shift toward a reactive state and produce pro-inflammatory cytokines, chemokines, reactive oxygen species, and inflammasome-associated mediators, thereby contributing to synaptic dysfunction and neuronal injury [23]. NF-κB signaling is one of the core transcriptional pathways regulating microglial inflammatory activation and has been widely implicated in AD pathogenesis [41]. In this study, single-nucleus transcriptomic analysis showed that NF-κB signaling activity was preferentially enriched in microglia, supporting microglia as a major cellular context for inflammatory pathway activation in AD. Moreover, CHUK and NFKB1, two key components of the NF-κB signaling axis, were identified as shared molecular targets linking dinotefuran-associated target profiles with AD microglial transcriptional alterations. These findings suggest that microglial NF-κB signaling may represent an important mechanistic bridge between dinotefuran exposure and AD-related neuroinflammatory responses.

Structural and experimental analyses further supported the potential involvement of dinotefuran in NF-κB-associated inflammatory signaling. Molecular docking and molecular dynamics simulations demonstrated stable interactions between dinotefuran and NF-κB-related proteins, including IKKα and NF-κB p50, suggesting that dinotefuran may potentially influence inflammatory signaling activity through interactions with key regulatory proteins. In addition, in vitro experiments showed that dinotefuran enhanced Aβ-induced inflammatory mediators and activation of NF-κB/NLRP3-associated signaling pathways. These findings are consistent with previous studies reporting that environmental toxicants can exacerbate neuroinflammatory responses through NF-κB-dependent mechanisms [42], [43]. Although further studies are required to determine whether direct molecular interactions occur in vivo, our results provide convergent computational and experimental evidence supporting the potential pro-inflammatory effects of dinotefuran in AD-related microglial activation.

From an environmental health perspective, the present study provides preliminary mechanistic insights into the potential association between neonicotinoid exposure and AD-related neuroinflammation. Increasing evidence suggests that environmental pollutants, including air pollutants, heavy metals, and pesticides, may contribute to neurodegenerative disease progression through chronic inflammatory and immune dysregulation mechanisms [44], [45], [46], [47], [48], [49]. However, most previous studies have primarily focused on traditional pesticide classes, whereas the neurodegenerative relevance of neonicotinoid pesticides remains insufficiently characterized. By integrating exposomic profiling, target-based pathway analysis, single-nucleus transcriptomics, structural modeling, and experimental validation, our study extends current understanding of neonicotinoid neurotoxicity beyond conventional neurobehavioral or oxidative stress-related effects. These findings highlight the importance of considering environmentally relevant pesticide exposure in AD-related neuroinflammatory research and provide additional insight into how environmental toxicants may influence neurodegenerative disease-associated inflammatory processes.

Several limitations should be acknowledged. First, functional validation was performed exclusively in BV2 microglial cells without in vivo confirmation. As BV2 cells cannot fully recapitulate the physiological characteristics of primary microglia and the complex AD brain microenvironment, further validation using primary microglia and appropriate animal models is warranted. Second, although seven representative neonicotinoid pesticides were initially evaluated through physicochemical characterization and target-based analyses, downstream mechanistic investigations focused exclusively on dinotefuran. Therefore, whether the observed pro-inflammatory effects are specific to dinotefuran or represent a broader feature shared by other neonicotinoid pesticides remains unclear. Third, molecular docking and molecular dynamics simulations only predicted potential interactions between dinotefuran and IKKα/NF-κB1, without direct biochemical binding confirmation. Further binding assays and target-specific genetic studies are needed to validate these proposed molecular interactions and their functional relevance. Fourth, the dinotefuran concentration used in this study (100 μM) was selected to investigate its potential toxicological mechanisms under experimental conditions. However, this concentration exceeds typical human environmental exposure levels. Future studies incorporating environmentally relevant concentrations and chronic low-dose exposure models are warranted to further evaluate the physiological relevance of our findings.

## Author contributions

Gaowa Baibujiafu: Writing - original draft, Investigation. Yuwan Lin: Methodology, Formal analysis. Lei Zhang: Software, Methodology. Lei Du: Writing - review & editing. Shan Yang: Methodology, Formal analysis. You'e Yu: Methodology, Formal analysis. Kaisai Ainiwaer: Methodology, Formal analysis. Jinjing Luo: Methodology, Formal analysis. Xiaobei Wang: Project administration, Funding acquisition, Writing - review & editing, Formal analysis, Conceptualization.

## Funding

This project was supported by the Key Laboratory of High Incidence Disease Research in Xinjiang (10.13039/501100004880Xinjiang Medical University), Ministry of Education (No. 2024B06); and Postdoctoral Research Startup Funding of Guangzhou City (No. 02018358).

## Declaration of competing interest

The authors declare that they have no known competing financial interests or personal relationships that could have appeared to influence the work reported in this paper.

## Appendix A Supplementary data

Table S1 Predicted target genes of representative neonicotinoid pesticides obtained from the TargetNet database.
